# Scrub typhus in the northern provinces of Vietnam: an observational study of admissions to a national referral hospital

**DOI:** 10.1093/trstmh/tru145

**Published:** 2014-11

**Authors:** Behzad Nadjm, Pham T. Thuy, Van D. Trang, Le Dang Ha, Nguyen V. Kinh, Heiman F. Wertheim

**Affiliations:** a Oxford University Clinical Research Unit, Hanoi, Vietnam; b Bach Mai Hospital, Hanoi, Vietnam; c National Hospital of Tropical Diseases, Hanoi, Vietnam

**Keywords:** *Orientia tsutsugamushi*, Scrub typhus, South East Asia, Vietnam

## Abstract

**Background:**

Scrub typhus is a common cause of fever in parts of South East and Southern Asia. Little is known about the disease burden in Vietnam.

**Methods:**

A 2-year observational study of scrub typhus at a tertiary referral hospital in northern Vietnam was carried out. Diagnosis was based on a single serological test in patients with suggestive clinical symptoms.

**Results:**

Scrub typhus was diagnosed in 3.5% (251/7226) of admissions. Cases occurred throughout the year, with incidence highest in the summer. Although complications were common, mortality was low (1.2%; 3/251).

**Conclusions:**

These data suggest that scrub typhus is common, with a seasonal distribution in northern Vietnam.

## Introduction

Scrub typhus, a zoonosis transmitted by trombiculid mites (known as ‘chiggers’) is caused by *Orientia tsutsugamushi* (Family: Rickettsiaceae), previously known as *Rickettsia tsutsugamushi*. Scrub typhus is a common cause of fever in parts of Asia. There have been reports of an increasing incidence of scrub typhus in this region.^1^ Predominantly an infection of rural populations, cases have been associated with clearing land, logging, military activities and rice farming.^2^ There are historic reports from the southern provinces of Vietnam,^3^ but few recent data, and to our knowledge no studies describing scrub typhus in the northern provinces of Vietnam.

We report results of a 2-year study into the frequency, clinical features and outcome of scrub typhus in patients admitted to a tertiary referral hospital in Hanoi, which received referrals from hospitals and health centres throughout northern Vietnam.

## Materials and methods

Adults and children admitted to the Infectious Disease Department of Bach Mai hospital (now the National Hospital of Tropical Diseases), between 2001 and 2003, had serological testing performed for scrub typhus by ELISA (Panbio, Melbourne, Australia) or Tsutsugamushi rapid test (SD Bioline, Kionggi, South Korea), if they presented with either fever with eschar, lymphadenopathy and rash or fever with no signs of pneumonia, urinary infection or other confirmed infections. A positive response (IgG/IgM by ELISA or IgG/IgM/IgA by rapid test) was considered a positive case of scrub typhus. Clinical features at admission, complications occurring during stay, treatment and outcome were recorded for all cases and descriptive statistics are presented in this study.

## Results and Discussion

**Figure 1.**
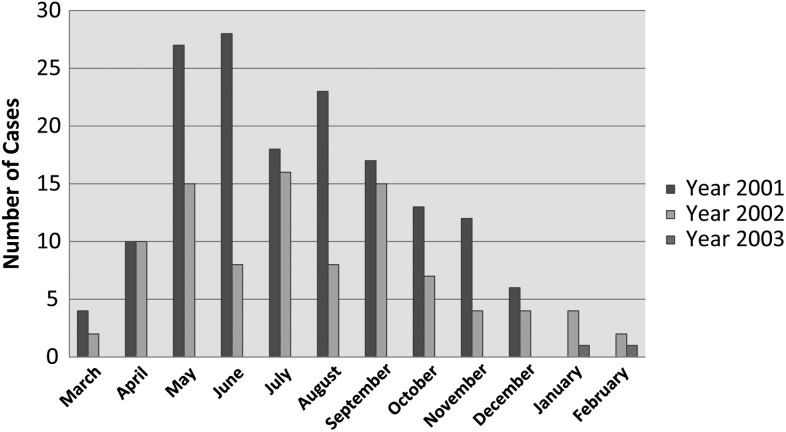
Frequency of scrub typhus cases per month from March 2001 to February 2003 in northern Vietnam.

Over the 2-year study, 749/7226 (10.4%) total admissions met the clinical criteria for testing; with 251 (3.5%) positive cases. The occupation of patients was recorded as farmer, student, teacher, housewife and ‘other’. Most patients with scrub typhus reported their occupation as farming (140/251; 55.7%), while patients from no other occupations were found to be at increased risk. Of note, military personnel were unlikely to attend this hospital as alternative medical facilities were used by this group. Peak incidence was reported to be in the fifth decade of life. Patients presented throughout the year, though incidence was highest in the summer months (Figure 1), coincident with rice farming activities. Although cases were found in all age groups, only 8 (8/251; 3.2%) confirmed cases were found in children (≤15 years). All patients had been resident in the northern provinces of Vietnam prior to the onset of illness and most patients reported a rural residence (183/251; 72.9%).

The most common clinical features, other than fever, were skin hyperaemia (85.6%; 215/251), headache (79.6%; 200/251), lymphadenopathy (61.7%; 155/251) and hepatomegaly (53.7%; 135/251). An eschar was found in 61.7% (155/251) of cases. Complications in patients were common and included: altered mental status (45/251; 17.9%), jaundice or hyperbilirubinaemia in (42/251; 16.7%) and pulmonary pathology in 39/251 (15.5%) cases. Most patients (136/232; 58.6%; no data for 19 patients) were treated with doxycycline, 10.3% (24/232) with chloramphenicol and 31.0% (72/232) with a combination of the two. There was often a delay in presentation leading to a median time from symptom onset to treatment of 10 days (interquartile range 8–12). Within 48 hours of starting antibiotic therapy 54.2% (136/251) of patients were afebrile. Mortality was low (1.2%; 3/251). The three deaths that did occur were in older individuals who presented with multiorgan failure that was felt to be directly attributable to scrub typhus.

These data are in-line with other reports, with low mortality expected in the group of treated individuals. The high proportion of patients presenting with a visible eschar must be interpreted in the context of using this finding as part of the inclusion criteria. In contrast, eschar was noted in only 7% (5/73) of children diagnosed with scrub typhus in southern Thailand.^4^ However, in our study, our patients were mainly adults. We noted some seasonal variation, with a drop in cases during the cool winter (December to March), which is in-keeping with the findings of other studies in other sub-tropical climates.^1^

The diagnosis of scrub typhus is not straightforward. Clinical and laboratory features are non-specific with overlap between scrub, other rickettsial infections, dengue fever and leptospirosis amongst others. Microbiological confirmation is also difficult. *O. tsutsugamushi* cannot be easily cultured, the sensitivity of PCR for the detection of DNA in blood is low, detection of DNA in punch biopsies of the eschar can be helpful, but is not usually possible for routine care. The gold standard for diagnosis requires paired sera for indirect immunoflourescence. Few data on serological cross-reactivity between *O. tsutsugamushi* and other infections are available. Cross-reactivity with *R. typhi* has been found in 18% of sera that tested positive for *O. tsutsugamushi*, but establishing whether this represents serological cross-reactivity or dual infection is difficult.^5^ A limitation of our data is that they do not meet these diagnostic standards nor was serology for other potentially cross-reactive pathogens performed. However, we believe that by restricting the population tested to those with clinically typical findings, our data are valid and suggest that *O. tsutsugamushi* is likely to cause significant morbidity in northern Vietnam. Indeed, given the high rate of pulmonary and CNS complications detected, it is possible that had patients admitted with pneumonia or meningo-encephalitis been tested for scrub typhus the incidence would have been higher.

Previous studies have demonstrated seasonal transmission of *O. tsutsugamushi* in more temperate climates with perennial transmission in tropical climates.^1^ Thus, our study may not be representative of the pattern of disease in the southern provinces of Vietnam, where the climate is tropical. Furthermore our study now represents quite old data and prospective studies using more rigorous diagnostics and systematic collection of data on potential risk factors are needed to describe the current epidemiology of scrub typhus across the whole of Vietnam.
